# Failure Diagnosis for Dental Air Turbine Handpiece with Payload Using Feature Engineering and Temporal Convolution Network

**DOI:** 10.3390/bioengineering11060555

**Published:** 2024-05-30

**Authors:** Yi-Cheng Huang, Po-Chen Chen

**Affiliations:** 1 Department of Mechanical Engineering, National Chung Hsing University, Taichung 40227, Taiwan; 2 Department of Mechatronics Engineering, National Changhua University of Education, Changhua 50074, Taiwan; yidajy2012@gmail.com

**Keywords:** dental air turbine handpiece, temporal convolution network, long short-term memory, convolutional neural network, failure classification

## Abstract

The internal mechanisms of dental air turbine handpieces (DATHs) have become increasingly intricate over time. To enhance the operational reliability of dental procedures and guarantee patient safety, this study formulated temporal convolution network (TCN) prediction models with the functions of causality in time sequence, transmitting memory, learning, storing, and fast convergence for monitoring the health and diagnosing the rotor and collet failure of DATHs. A handpiece mimicking a dentist’s hand load of 100 g was employed to repeatedly mill a glass porcelain block back and forth for cutting. An accelerometer was employed to capture vibration signals during free-running of unrestrained operation of the handpiece, aiming to discern the characteristic features of these vibrations. These data were then utilized to create a diagnostic health classification (DHC) for further developing a TCN, a 1D convolutional neural network (CNN), and long short-term memory (LSTM) prediction models. The three frameworks were used and compared for machine learning to establish DHC prediction models for the DATH. The experimental results indicate that, in terms of DHC predicted for the experimental dataset, the square categorical cross-entropy loss function error of the TCN framework was generally lower than that of the 1D CNN, which did not have a memory framework or the drawback of the vanishing gradient problem. In addition, the TCN framework outperformed the LSTM model, which required a longer history to provide sufficient diagnostic ability. Still, high accuracies were achieved both in the direction of feed-drive milling and in the gravity of the handpiece through vibration signals. In general, the failure classification prediction model could accurately predict the health and failure mode of the dental handpiece before the use of the DATH when an embedded sensor was available. Therefore, this model could prove to be a beneficial tool for predicting the deterioration patterns of real dental handpieces in their remaining useful life.

## 1. Introduction

Dental air turbine handpieces stand as the most prevalent medical apparatus employed by dentists in the treatment of teeth. The primary mechanical operation of the aforementioned equipment entails the infusion of high-pressure air into the head to activate microturbine blades, which in turn drive the rotation of bearings that power a tooth drill. Prior to or during treatment, dentists are unable to discern the state of health (SOH) or detect any damage within the internal components of a dental handpiece. Due to the high rotational velocity of a dental handpiece, its internal bearings progressively deteriorate and incur damage over prolonged usage. As a result, ceramic balls are unable to maintain stable operation along their confined track, leading to heightened internal friction within the machine head’s housing and an increase in temperature. The heat is transferred to the bur via the rotor, thereby elevating the temperature of the bur. When the temperature of the bur’s milling contact surface exceeds 42.5 °C [1], it has the potential to inflict irreversible damage to teeth. Furthermore, at 52 °C, it may lead to pulp necrosis. The temperature rise causes the rotor collet to loosen. This may cause unbalanced vibration in the bur and even cause it to fly out of the DATH. In addition, the vibrations generated by the bur induce discomfort to the patient and result in an uneven surface on the tooth.

The mean clinical force applied to the DATH with a bur in the chuck was reported as 99.3 g, substantiating the range 50–150-g range [2]. A high-speed handpiece with cutting efficiency according to the bur eccentricity with a constant weight load of 0.9 N for 15 s was also studied in [3]. An experimental custom-made device was used to hold the high-speed handpiece and specimen in their cutting test. In their in vitro study, the user’s loading configuration was set to apply a load using the handpiece within the patient’s oral cavity. The handpiece and the specimen were fixed during the test. Therefore, this loading to the DATH was mimicked in this study.

Zakeri and Arzanpour [4] utilized an accelerometer and a laser Doppler vibrometer to capture vibration signals while operating a dental handpiece. Through the comparison of spectrograms between the handpiece operating without load (free running) and under load (milling teeth), the aforementioned authors discerned a decrease in vibration frequency during milling. As the diagnostic perspective point of view, the vibration information from the free running after the milling process will be applied in this study after milling the specimen with a load of 0.9 N. Wei, Dyson and Darvell [5,6] used different operating conditions for a DATH to generate an experimental group and a control group. Dental handpieces were used according to pre-established testing procedures until their bearings failed, through which they analyzed factors affecting the service life of the bearing. In [6], they noted that the ball bearing’s failure typically stemmed from faults in the cage (the non-metallic component of the bearing). Upon bearing failure, the cage incurs significant damage due to the instrument operating under load and the presence of corrosion during high-pressure sterilization. While it remains uncertain whether wear and fracture of the cage directly cause bearing faults, damage to the cage undoubtedly impedes the stable functioning of the ball within the raceways, thus leading to damage of the ball and raceways after relatively few rotations. From our in-laboratory experimental experiences, the failure of the cage, which may be caused by ceramic ball bearings, will unbalance the rotor. The unbalanced rotor can come into contact with the housing and lead to frictional force. A picture of the damaged rotor showed a brown color, frictional marks, and corrosion patterns. Consequently, frictional heat is generated, resulting in the formation of a heat-affected zone. When a dentist uses the handpiece, this heat is inevitably transferred to the dental drill. Such an issue should be prevented and diagnosed in advance in vivo in dental operations.

The advent of artificial intelligence has been accompanied by diverse maintenance strategies, and the assessment of machine health has become a significant focus. Among these methods, the support vector machine (SVM) has garnered attention due to its effective classification capabilities and minimal training and testing time requirements. Notably, SVM excels in classifying diverse classes even with limited training and testing data, reducing computational load [7]. However, the SVM algorithm has its limitations. Optimal parameter selection for various kernel functions and managing cost parameters are crucial for SVM performance since each classification problem corresponds to an optimal parameter set. Additionally, SVM’s diagnostic accuracy may suffer if training data for different classes are ambiguous or poorly differentiated.

Today, the rapid technological evolution of hardware and software has opened up avenues for research leveraging artificial intelligence [8], image processing, computer vision, and machine learning.

The application of the convolutional neural network (CNN) is the main research area of artificial intelligence, especially in image diagnosis. In [9], a CNN was used for fault identification and classification in gearboxes. In their study, the vibration signals were preprocessed using statistical measures from the time-domain signal, such as the standard deviation, skewness, and kurtosis of the raw data. In the frequency domain, they divided the spectrum obtained with FFT into multiple bands, and calculated the root mean square (RMS) value for each one. So, the vibration energy, maintaining the shape of the associated spectrum peaks, could be acquired. The diagnosis accuracy indicated that the proposed CNN approach was more reliable than the SVM. In [10], a deep-structure CNN was proposed as a method for diagnosing bearing faults by using vibration signals directly as input data. They transferred the vibrational signal into a vibration image, and then, learned using a CNN without requiring any feature extraction techniques. Their method was for an automatic fault diagnosis system under noisy environments. In [11], a 1D CNN was used as a classifier with a compact architecture configuration for real-time bearing fault detection and monitoring. The motor vibration and current datasets were experimentally validated.

The long short-term memory (LSTM) network has demonstrated its capability to transfer memory and learn from time-series data. Current research has applied this network in various areas, including fault prediction, prognostics, health management, system status monitoring, and predictive maintenance. Recent studies have developed LSTM prediction models specifically tailored for monitoring the health and degradation of dental air turbine handpieces (DATHs). In one such study [12], a handpiece was utilized to perform cutting operations on a glass porcelain block repeatedly. An accelerometer was employed to capture vibration signals during the free-running mode of the handpiece, enabling the identification of characteristic frequencies in the vibration spectrum. The gathered data were then used to establish a health index (HI) for constructing predictive models. In that research, the cutting force applied to the DATH was not studied. The LSTM model can be a useful tool for predicting the degradation trajectory of real dental handpieces. In addressing the precise classification of health states, the development of feature engineering, based on the principles of data mining to expand the dimensions of raw data, proves advantageous. Subsequently, the selection of optimal combinations of characteristic features from the expanded dataset ensues. Data preprocessing has emerged as a more resilient and sophisticated method for diagnosis, especially with the rise of artificial intelligence. In [13], a systematic assessment was conducted on generic convolutional and recurrent architectures for sequence modeling. Results from the described generic temporal convolutional network (TCN) architecture convincingly outperformed the LSTM and gated recurrent unit (GRU) on many test datasets, such as the sequential MNIST. The advantages of using TCNs for sequence modeling include parallelism, flexible receptive field size, stable gradients, low memory requirement for training, and variable-length inputs [13]. A framework was proposed by using time empirical mode decomposition (EMD) and a TCN for remaining useful life (RUL) estimation for the bearing failure in [14]. They decomposed the original signal data by EMD and expanded the data to 12 dimensions. Then, the processed datasets were used to train the TCN. Their results indicated that LSTM and CNN struggled to capture the degrading trend exactly but EMD-TCN was effective in prediction by combining the historical condition and convolution. In [15], the RULs of the bearing datasets were predicted by a TCN by conducting vibrational signal segmentation and using statistical features as the input of the TCN. The RUL results showed the proposed TCN’s superiority when the random forest (RF), LSTM network, and gated recurrent unit (GRU) were compared. Modifications of the TCN’s structure can be found in [16,17] by using the Squeeze excitation block and residue block of the TCN for reducing the training time, prevention of overfitting, and improving model accuracy.

This paper deploys the TCN architecture for feature learning and performing accurate predictions with different fault diagnoses for the DATH. Notable advantages and improvements are observed with the TCN model’s prediction accuracy in this research compared to the 1D CNN and LSTM approaches. The experimental results with the applied load on the DATH suggest future practical dental implementations and pilot studies of the proposed predictive model in clinical settings. The rest of the paper is organized as follows. In Section 2, the theoretical background for feature engineering with feature extraction and selection are described. The fundamentals of the TCN with nine diagnostic health classification (DHC) labels for the DATH are introduced. Section 3 presents the experimental setup and results by using feature engineering followed by 1D CNN, LSTM, and TCN. Section 4 provides a discussion. Some concluding remarks and recommendations for future work are summarized in Section 5.

## 2. Theoretical Background of Feature Engineering, TCN, CNN, and LSTM

### 2.1. Feature Engineering, Feature Extraction, and Feature Selection

**Figure 1:**
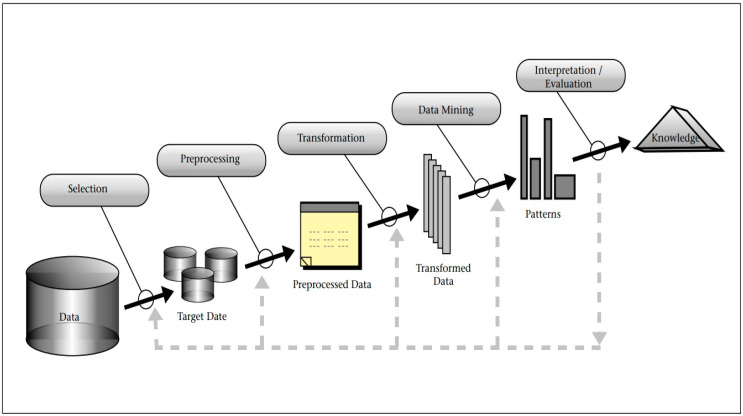
Overview of steps for composing the KDD [19].

Knowledge Discovery Databases (KDDs) [18], as illustrated in Figure 1, pertain to a paradigm for data consolidation encompassing feature extraction and feature selection. The five tiers of sensor fusion delineated in the Dasarathy model [19] are as follows. Initially, spanning level one to level three, input and output data undergo data-driven feature analysis, followed by feature selection preceding feature extraction, culminating in decision-making at level four and five. Lately, numerous researchers have employed KDDs or data fusion for diagnosing mechanical faults. Data preprocessing has emerged as a more robust and sophisticated approach to diagnosis, especially given the ascendance of artificial intelligence. The fundamental principle of feature engineering is to discern the most discriminative features from raw data through systematic operations. In [20], the raw vibration data were segmented followed by using feature extraction methods in an attempt to extract representative quantitative values that could reflect the ball screw system’s behavior properties. Feature extraction reduced the data quantity and complexity, and then, it was followed by selection based on Fisher criteria or principal component analysis (PCA). Machine learning methods via self-organizing maps (SOMs), Mahalanobis distance, and Gaussian mixture model (GMMs) were used for ball screw fault diagnosis. In this study, the concept of KDD following a systematic feature extraction procedure is adopted.

First, statistical features consisting of the mean, root mean square (RMS), kurtosis, skewness, crest factor, variance, and standard deviation are extracted in each section of the time domain. In the second step, the vibration raw data (denoted as xi) in the time domain with a sampling rate of 12.8 kHz are transformed into power spectrum density amplitude (PSD-Amp) and PSD shape (PSD-Shape) in the frequency domain. Four statistical features, namely, mean, standard deviation, skewness, and kurtosis are extracted in each section of PSD-Amp and PSD-Shape. They are extracted from both the time and frequency domains. Last, the vibrational data in the frequency domain are divided into 256 sectors, with a width of 25 Hz for each frequency sector. Then, the frequency bandwidth goes up to 6400 Hz. Within each section, the mean value of the amplitude sum is computed as a feature.

Table 1 and Table 2 present the six features in the time domain and the eight features calculated from the frequency-domain amplitudes for each section with divisions every 25 Hz. In addition, the 256 sections featured in the frequency domain are gathered with the above 14 features. Thus, a total of 270 (6 plus 8 and plus 256) extracted features are fed for feature selection. Typically, outliers for each feature type are eliminated using the Z-score formula to prevent their influence during algorithm training. In this study, the Z-score was not applied for the sake of testing the robustness of the developed 1D CNN, LSTM, and TCN. In the feature selection step, the most discriminative feature among all features is determined using Fisher’s criteria. The score obtained from Fisher’s criteria serves as an index of the degree of distinctiveness. Each feature can be given a Fisher score with two different diagnosed items, such as the normal DATH and the failure of the DATH’s rotor. Subsequently, the two, three, or more highest scoring features are selected as training data for the algorithm. In this study, five features were selected. Before all the selected features were fed as the input items of the NNs (1D CNN, LSTM, and TCN), the feature data were all scaled to the same order using a normalization process to reduce the computation complexity.

### 2.2. Fundamentals of TCN

TCN [13] is a kind of sequential prediction model that is designed to learn the hidden temporal dependencies within input sequences. It takes the sequence of the input, and the corresponding parameters as the expected outputs. The architecture of TCN is characterized with dilated convolution, causal convolution, residue connection, and a fully connected layer. Some details about TCN are as follows.

First, the dilated convolution allows a receptive field with more wide convolutions among the hidden layers, since the filter serves as the function for the feature map. As the number of hidden layers increases, introducing a dilated factor enlarges the receptive field in the feature extraction domain.

The dilated factor $d_{i}$ increases with the number of hidden layers n, as in Equation (1). For a sequence input X and a filter $f\left(i\right)$, Equation (2) presents the dilated convolution operation $F\left(s\right)$ on data point s of the sequence, where s=0,1,…,T, where $f\left(i\right)$ is the filtering value, and k is the size of filter in dilated convolutions. The conventional discrete convolution * is simply the 1-dilated convolution. Here, we refer to ∗$d_{i}$ as a dilated convolution or a $d_{i}$-dilated convolution. $N_{Stack}$ is the stacked number of dilated causal convolution layers in one TCN layer, it is usually set as 1. The relationships between the receptive field and the filter size, stacked number, and dilated factor is shown in Equation (3).

(1) $$d_{i}=2^{n}$$

(2) $$F\left(s\right)=\left({X*}_{d_{i}}f\right)(s)=\sum_{i=0}^{k-1}f\left(i\right){\cdot X}_{s-d_{i}}$$

(3) $$R_{Field}=1+2\left(k-1\right)\cdot N_{Stack}\cdot \sum_{i=0}^{n}d_{i}$$

**Figure 2:**
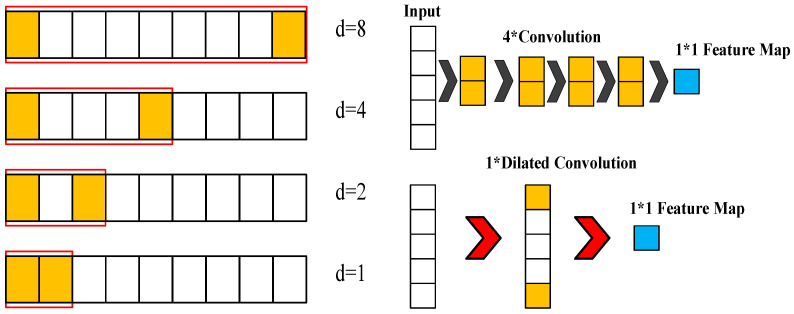
Illustration for the efficiency of using the dilated convolution with *d* = 4 (**upper right**) compared with *d* = 1 (**lower right**) for the same input receptive field of 5 neurons.

**Figure 3:**
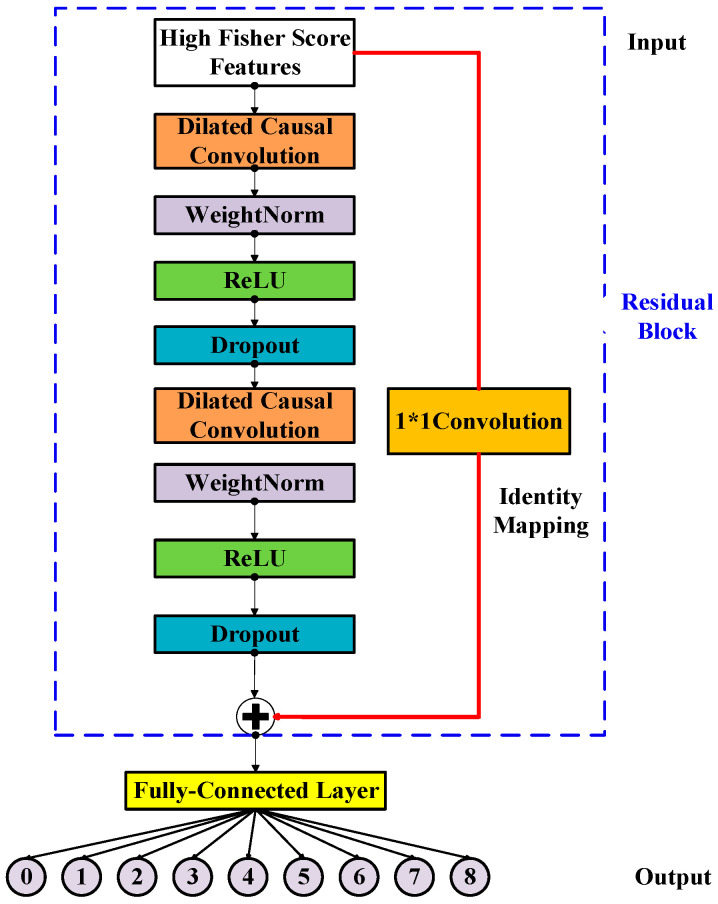
The architecture elements of a TCN residue block with input features via feature engineering.

**Figure 4:**
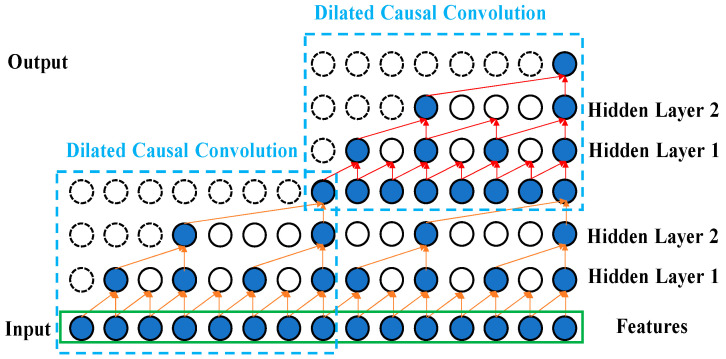
Illustration for the two dilated causal convolution layers.

Secondly, the causal convolution can deal with the sequenced vibrational signal and prevent the future data information being convoluted with past information. This reduces the computation time. Figure 2 illustrates the efficiency of using the dilated convolution with *d* = 4 (upper right) compared to with d = 1 (lower right) for the same input receptive field of five neurons. The inputs are the selected Fisher score features processed by feature engineering. Causal convolution allows the TCN, 1D CNN, and LSTM models to make predictions on continuous vibrational data for performing the diagnostics of the DATH’s health. The residue connection was proposed in [21]. In Figure 3, this network structure preserves the initial input value by a feedforward loop of identity mapping together with two hidden layers where the vanishing gradient problem is avoided. As shown in Figure 4, two dilated causal convolution layers associated with the identity mapping form the architecture elements of a TCN residue block.

### 2.3. Fundamentals of Convolutional Neural Network

**Figure 5:**
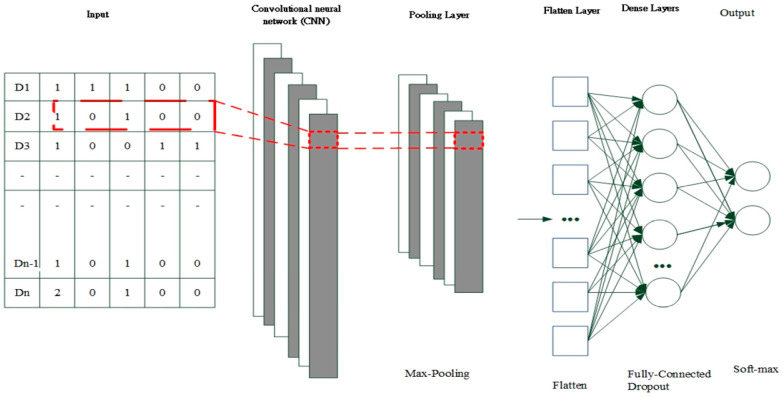
The architecture of CNN.

CNNs hold promise for processing sensor measurement data and deriving intricate spatial features. CNN architectures typically encompass a convolutional layer, a pooling layer, a fully connected (FC) layer with dropout, and a softmax layer for classification (see Figure 5). Within the convolutional layer, convolutional filters are leveraged to derive features from the input data, thereby facilitating feature extraction and mapping. Subsequently, an activation function is applied to yield the feature map for the subsequent layer. The output of the convolution filter is computed utilizing the following formula:

(4) $$z^{l}=w^{l}*{cl}^{l-1}+b^{l}$$

(5) $${cl}^{l}={ac(z}^{l})$$

Where ∗ denotes the convolutional operation, l is the layer number in the network, and ${cl}^{l-1}$ and ${cl}^{l}$ are the input and output of the convolution filter, respectively. Moreover, $ac\left(.\right)$ is the activation function, $z^{l}$ is the input of the activation function, $w^{l}$ is the weight matrix of the convolutional filter, and $b^{l}$ is the additive bias vector.

Within the pooling layer, the output of the convolutional layer undergoes downsampling by an appropriate factor to diminish the resolution of the feature map. Among the prevalent methods, average pooling and maximum pooling stand out, with the latter being employed in this study. The formula for maximum pooling is delineated as follows:

(6) $${cl}^{l}=down({cl}^{l})$$

where down(·) represents the downsample function for maximum pooling.

FC layers integrated with dropout are employed to facilitate training in deep neural networks. During each training batch, half of the feature detectors are disregarded, leading to a significant reduction in overfitting. This technique diminishes the interdependence among feature detectors, thereby preventing some detectors from becoming overly reliant on others to perform their functions.

Upon applying the softmax operation, the summation of the probability distributions of the outputs from the final layer equates to 1. For instance, in a classification scenario featuring two categories, where the input is an image signal, the softmax activation function outputs the probabilities associated with the image belonging to the aforementioned categories. The cumulative sum of these probabilities amounts to 1.

(7) $${S(fl)}_{i}=\frac{e^{{fl}_{i}}}{\sum_{j=1}^{C}e^{{fl}_{j}}}$$

In Equation (7), ${S(fl)}_{i}$ is in the range (0, 1), which is required for this value to be a probability. The fl input vector for the softmax function is given as (${fl}_{0}$, …, ${fl}_{c}$), with elements ${fl}_{i}$. The standard exponential function is applied to each element of the input vector. Division by $\sum_{j=1}^{C}e^{{fl}_{j}}$ ensures that the sum of the softmax output values is 1; this term is the normalization term. C is the number of classes in the multiclass classifier [22].

### 2.4. Fundamentals of Long Short-Term Memory

**Figure 6:**
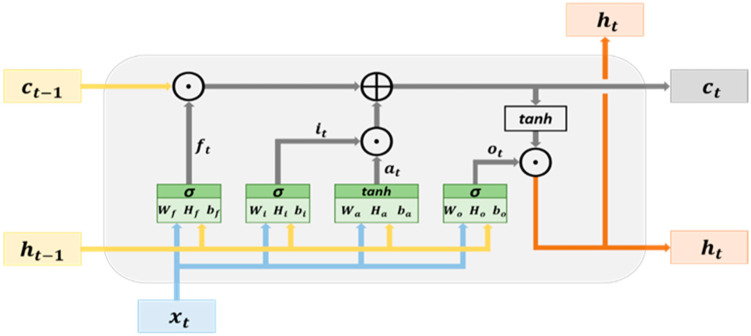
The architecture of LSTM memory cells.

An LSTM network possesses a memory structure comprising memory cells that facilitate the addition and retention of information as the time series progresses, effectively addressing the vanishing gradient problem. Figure 6 delineates the fundamental architecture of an LSTM network. The cell state serves as a repository for storing and transmitting memory, thereby ensuring that the information contained therein is only susceptible to writing or deletion. Without external influence, the aforementioned information remains unaltered. The parameter $x_{t}$ signifies the input data at time t, while $h_{t-1}$ denotes the hidden state at time t−1. The cell state at time t−1 is denoted as $C_{t-1}$, which undergoes modification to yield the current cell state $C_{t}$ within the hidden layer at time t.

**Figure 7:**
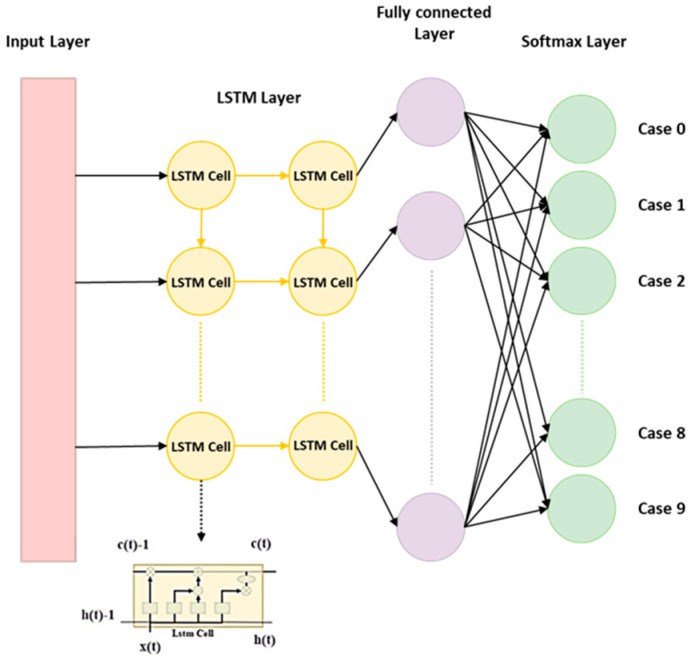
The architecture of LSTM for classification.

The hidden layer within an LSTM network comprises an input node, denoted as $a_{t}$, and three controlled gates, namely, $f_{t},i_{t},\;and\;o_{t}$. These variables $a_{t}$, $f_{t},i_{t},\;and\;o_{t}$ are computed using Equations (1)–(4), respectively. The input node $a_{t}\;$plays a role in updating the cell state, while the controlled gates are responsible for determining whether to permit the passage of information through them. Specifically, the controlled gates consist of the forget gate, input gate, and output gate. The forget gate $f_{t}\;$is instrumental in deciding which information from the previous cell states ($c_{t-1}$) may pass through it. On the other hand, the input gate ($i_{t}$) dictates which information from the input nodes $a_{t}\;$can pass through it. The vectors (information) that traverse through the input gate contribute to updating the cell state through element-wise addition with the vectors from the forget gate, ultimately generating the cell state at time t, denoted as $c_{t}$. This computation process is expressed in Equation (5). Moving forward, the output gate determines which information from the current cell state $c_{t}$ can pass through it. The information vectors that pass through the output gate contribute to the hidden state at time t, denoted as $h_{t}$, serving as the output vectors of the ongoing hidden layer. The computation method for $h_{t}\;$is elucidated in Equation (6). Moreover, the cell state $c_{t}$ and hidden state $h_{t}\;$obtained at time t are propagated to the hidden layer at time t+1. This iterative process, progressing with the time series, facilitates the transmission and acquisition of memory, contributing to the learning process.

(8) $$a_{t}=tan\;h(W_{a}x_{t}+H_{a}h_{t-1}+b_{a})$$

(9) $$f_{t}=\sigma (W_{f}x_{t}+H_{f}h_{t-1}+b_{f})$$

(10) $$i_{t}=\sigma (W_{i}x_{t}+H_{i}h_{t-1}+b_{i})$$

(11) $$o_{t}=\sigma (W_{o}x_{t}+H_{o}h_{t-1}+b_{o})$$

(12) $$c_{t}=(f_{t}⊙c_{t-1})⊕(i_{t}⊙a_{t})$$

(13) $$h_{t}=o_{t}⊙tan\;h(c_{t})$$

where W and H represent the weights, b denotes the bias, ⊕ is the symbol for element-wise addition, ⊙ is the symbol for element-wise multiplication, tan h denotes the hyperbolic tangent, and σ represents the sigmoid function. The parameters tan h and σ represent activation functions. The current study adopted the LSTM structure as shown in Figure 7. The structure is used to predict the health status of the dental handpiece with 9 classifications.

### 2.5. Summary of the Framework of TCN, 1D CNN, and LSTM Prediction Models

**Figure 8:**
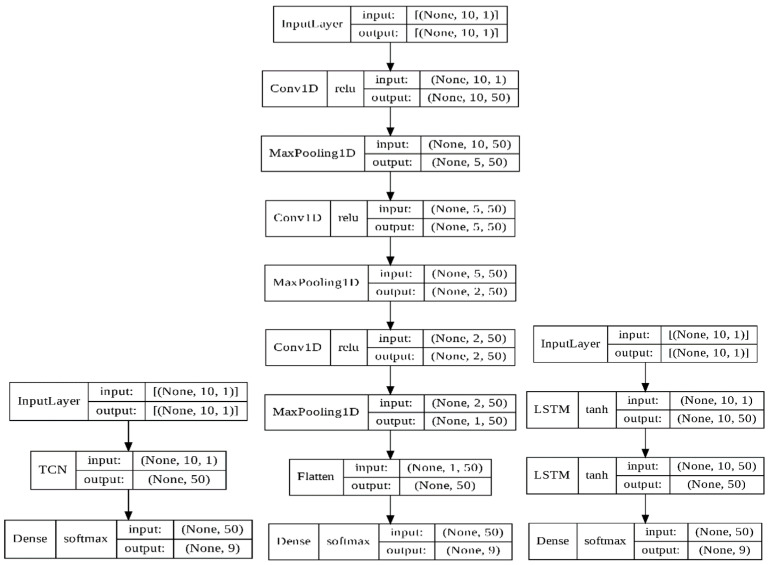
Frameworks for the TCN, 1D CNN, and LSTM.

Table 3 and Figure 8 detail the parameters of the TCN, 1D CNN, and LSTM by Keras. The notation ‘none’ in Table 3 indicates that the input and output dimensions were changed in the 1D CNN and LSTM models’ computations.

## 3. Experiments Results

### 3.1. Experimental Setup and Milling

**Figure 9:**
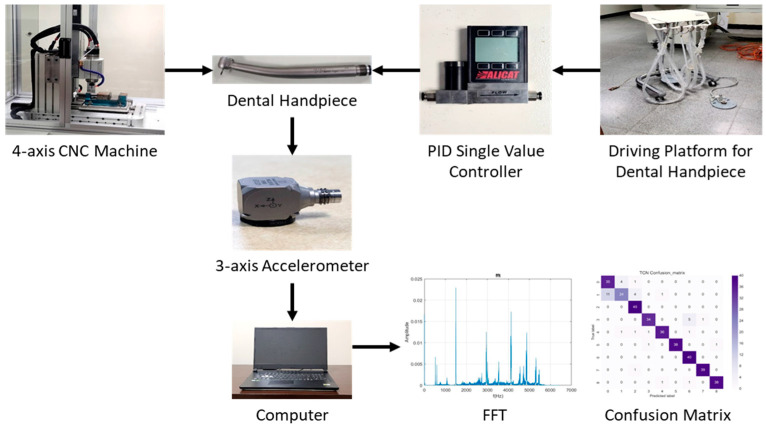
The experimental setup for the failure diagnosis of the DATH’s rotor and collet by milling process.

**Figure 10:**
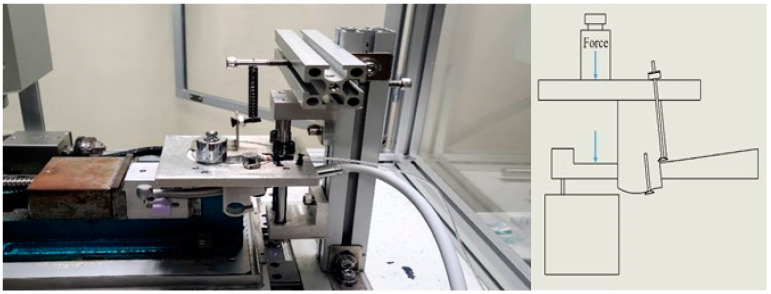
Picture of the DATH, glass porcelain block (purple color), attached accelerometer, and applied 100 g force, as illustrated on the right.

**Figure 11:**
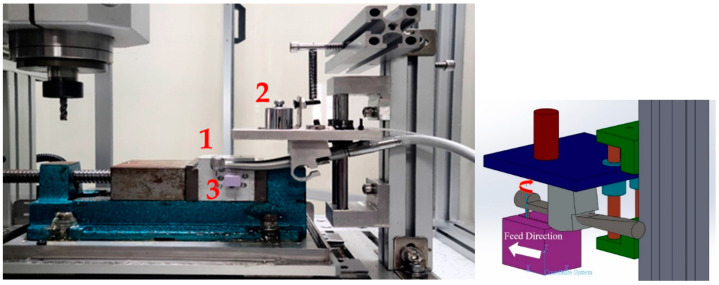
Picture of the DATH (no. 1), 100 g block, glass porcelain block (no. 3), and illustrated coordination for the feed direction (the feed direction is defined as being in the *Y*-axis) and spindle rotation.

The experimental apparatus comprised a dental device driving platform, a dental air turbine handpiece, and a four-axis table CNC machine (refer to Figure 9). The dental air turbine handpiece (Tiger101-3T4, manufactured by Thunder, Tiger group, Taichung, Taiwan) consisted of a machine head, hand grip, and handpiece connector. The carbide bur utilized had a diameter of 1.6 mm and a total length of 16 mm. This investigation employed a pressure control transducer (Alicat Scientific, Inc., Tucson, AZ, USA) PC-Series proportional–integral–derivative (PID) single-valve pressure controller to regulate the drive air pressure. An RS-232 signal line was interfaced with the Flow Vision SC software program running on a personal computer. Within the software interface, diverse parameters pertaining to the pressure control valve were configured to ascertain the positioning of said valve and the input voltage. Adjustments were made to the input voltage to manage the set point. A laboratory four-axis desktop computer numerical control (CNC) machine was used to mimic the cutting motion of a human operating on the dental handpiece with a payload of 100 g. The apparatus was utilized to manipulate the dental handpiece and a glass ceramic block for the purpose of conducting a cutting experiment. The material used for cutting was a glass ceramic tempered with IPS Empress CAD of SiO_2_-Al_2_O_3_-K_2_O material with a bending strength of 360 MPa. A triaxial accelerometer (8688A5, Kistler, Winterthur, Switzerland, sensitivity: 1000 mV/g) was glued to a jig. NI cDAQ-9174 and NI 9230 instruments were employed for the measurement and capture of the vibration signals emitted by the handpiece during unrestricted operation. The acquired data were transferred to and archived in LabVIEW. The sampling frequency was configured to 12.8 kHz. The experimental arrangement is depicted in Figure 10 and Figure 11.

**Figure 12:**
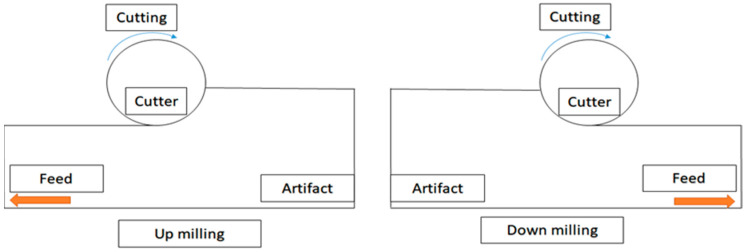
Schematics of up-milling (**left**) (used in this study) and down-milling (**right**).

**Figure 13:**
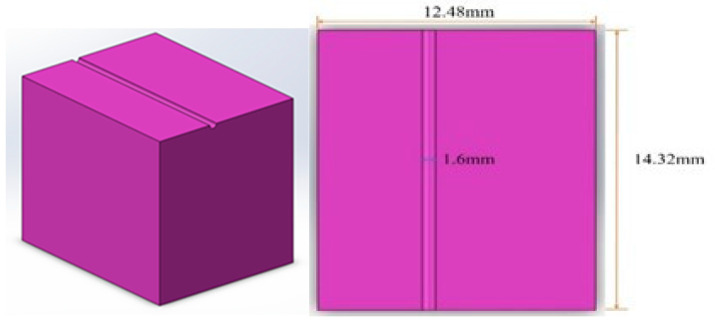
Schematic of the cutting path for one cut.

Figure 12 shows the schematics of up-milling used in this study. The cutting path for one cut is illustrated in Figure 13. The cutting conditions were as follows: the feed rate was set as 15, 30, 45, and 60 mm/min, with different air pressures of 30, 40, and 50 psi. The cutting width was 0.16 mm until the glass ceramic was cut to a depth of 0.1 mm, and the cutting path was 14.32 mm. The cutting process involved 10 repetitions of linear up-milling. Following the completion of the 10 cutting paths within a cycle, the vibration signals emitted by the dental handpiece during free-running operation were captured 5 s before and after the cutting cycle. Thus, free-running vibration signals were acquired for data analysis. Apart from mill wear, a new tooth drill (based on multiple experimental trials) could typically be utilized for 20 cycles. Subsequently, based on visual inspection outcomes, the tooth drill was replaced.

### 3.2. Free-Running Vibration Signals

**Figure 14:**
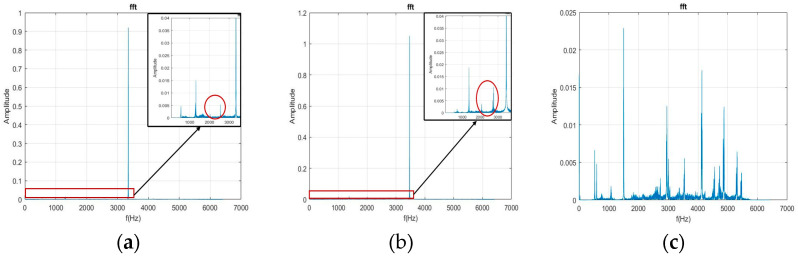
Plots of the FFT for healthy (**a**), damaged collet (**b**) and damaged rotor (**c**) DATH using 30 psi pressurized air.

**Figure 15:**
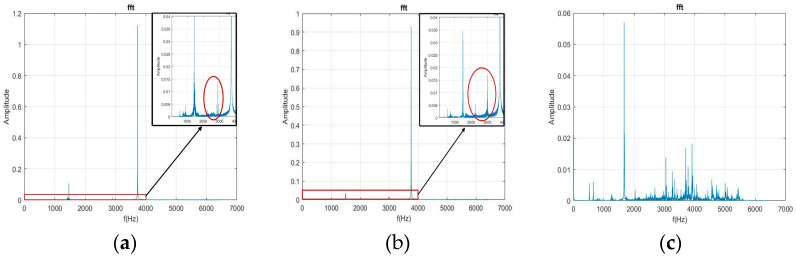
Plots of the FFT for healthy (**a**), collet failure (**b**), and rotor failure (**c**) DATH using 40 psi pressurized air.

The current study first used the FFT to transform the free-running vibration signals before and after the milling process for 5 s. In Figure 14a,b and Figure 15a,b, the healthy and collet failure signals exhibit significant characteristic frequency (CF) when the pressurized air is 30 psi and 40 psi. In Figure 14c and Figure 15c, the frequency distribution for the rotor failure of the DATH exhibits strong peaks over the whole frequency domain. This was caused by the effect of a bad bearing on the rotor. The unbalanced rotation reduced the value of the CF and rendered the energy of the pressurized air to the rotor’s in broad-band vibration. When Figure 14a,b are compared, some peak frequencies (lower than the CF) can be observed but they are hard to classify. These phenomena are maintained in Figure 15b,c. Therefore, it is not easy to verify the differences between the healthy, failed rotor, and failed collet dental handpieces.

### 3.3. Classification Results by Steps of Feature Engineering and Prediction Models

#### 3.3.1. Feature Selection

The raw data of the vibration signals were acquired, and then, processed, as shown in Table 1 and Table 2. Fisher’s criteria were used to select the significant features from the time and frequency features that could be used to distinguish the healthy, damaged rotor, and damaged collet DATH by following the TCN, 1D CNN, and LSTM classification models, since using all the extracted features for assessing the prognostic-diagnosis health model is unfeasible and inefficient. Some extracted features have a low contribution in classification models. Therefore, the Fisher criterion score (Equation (14)) was selected to determine significant features from the feature vectors. Each feature had its own score, which was calculated as follows:

(14) $$Fisher\;score=\frac{\mu_{1}-\mu_{2}}{{\sigma_{1}}^{2}+{\sigma_{2}}^{2}}$$

where *μ* and σ indicate the mean and standard deviation, respectively, of the same class, and 1 and 2 represent classes 1 and 2, respectively.

**Figure 16:**
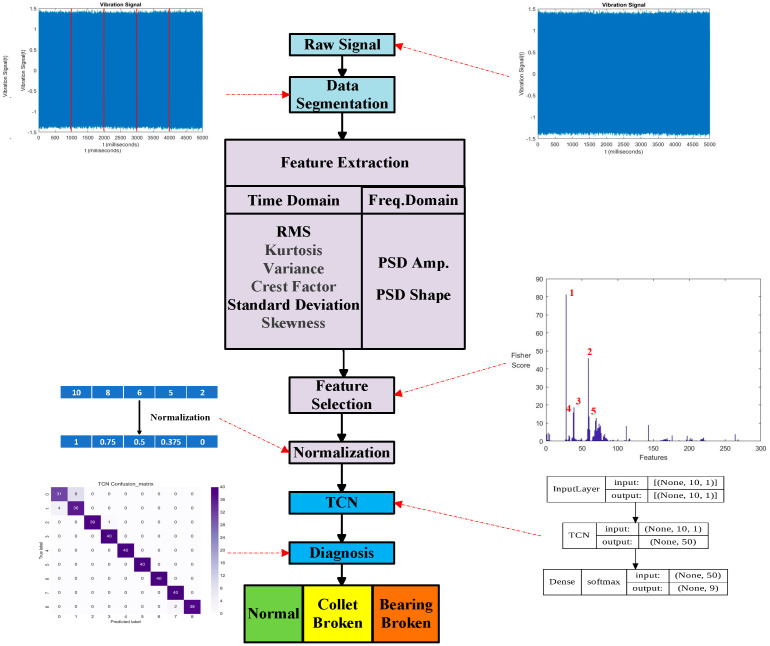
Illustration of the diagnosis prediction by steps of feature engineering and use of TCN classification model.

Figure 16 illustrates the diagnosis prediction by the steps of feature engineering and use of the TCN classification model. Feature selection was performed and followed by normalization. Normalization is essential for all the input of the TCN, 1D CNN, and LSTM. Table 4 displays the top five features from the *X*, *Y*, and *Z* axes’ signals from the triaxial accelerometer acquired during free running before cutting. The notation N30, D30, and B30 indicates the normal, damaged collet, and damaged bearing DATH, using 30 psi pressurized air. The top five features selected from the *X*, *Y*, and *Z* axes were different. They were based on comparing a normal DATH with a damaged collet DATH, damaged collet DATH with damaged bearing DATH, and normal DATH with damaged bearing DATH at 30, 40, and 50 psi.

As an example of the vibrational features in the *Y*-axis, the feature numbers 1, 2, 3, 5, 59, 62, 64, 66, 70, and 264 denote the significant features of RMS, variance, standard deviation, and kurtosis in the time domain, and the frequencies ranging from 1300 Hz to 1325 Hz, 1375 Hz to 1400 Hz, 1425 Hz to 1450 Hz, 1475 Hz to 1500 Hz, and 1575 Hz to 1600 Hz, and the power amplitude spectrum density. These 10 features were selected among the nine classes. The eight feature numbers selected from the *z*-axis were 1, 2, 3, 5, 62, 67, 70, and 264. They stand for the RMS, variance, standard deviation, kurtosis, frequencies ranging from 1375 Hz to 1400 Hz, 1500 Hz to 1525 Hz, 1575 Hz to 1600 Hz, and 1575 Hz to 1600 Hz, and the power amplitude spectrum density. Compared with the selected features of the *Y*-axis and *Z*-axis, 25 features were selected for the *X*-axis. Many diverse features were selected, which caused a low prediction accuracy.

The most focused features should be in the *z*-axis (axial direction of the gravitational force acting on the DATH and the payload). The other sensed vibrational signals will be in the *X*-axis and *Y*-axis (radial direction of the DATH’s spindle). However, the second-most focused features were observed in the *y*-axis, since the acquired vibrational signals were aligned with the feed-drive direction. So, when the pressurized air was excited, the constraint of the designed fixture (a four-bar linkage planar motion with an elastic spring in the Y-direction), as displayed in Figure 10 and Figure 11, should bear the sensed signals more solidly than the ones in the X-direction.

The total numbers of the top 5 selected features for the *X*-axis, *Y*-axis, and *Z*-axis free-running signals after the milling, were 33, 9, and 12, respectively. Experimental results coincided with the free-running before cutting. Therefore, the acquired vibration signals in either the *Y*-axis or the *Z*-axis were preferred.

#### 3.3.2. Model Prediction Results of TCN, 1D CNN, and LSTM

In this experiment, before and after each cutting the vibrational data were acquired for 5 s. The total milling process was conducted 40 times. There are 200 data for one data cell. The pre-cutting free-running data were grouped as one data cell, while the after-cutting free-running data were grouped as another data cell. In Table 5, as an example, there are 1800 data (nine cells) to be diagnosed. Such a dataset will be predicted by one of the TCN, 1D CNN, and LSTM models. The number of selected features in Section 3.3.1 will be the input number for the TCN, 1D CNN, and LSTM models. The parameters and frameworks of the TCN, 1D CNN, and LSTM models are stated in Table 3 and Figure 8.

**Figure 17:**
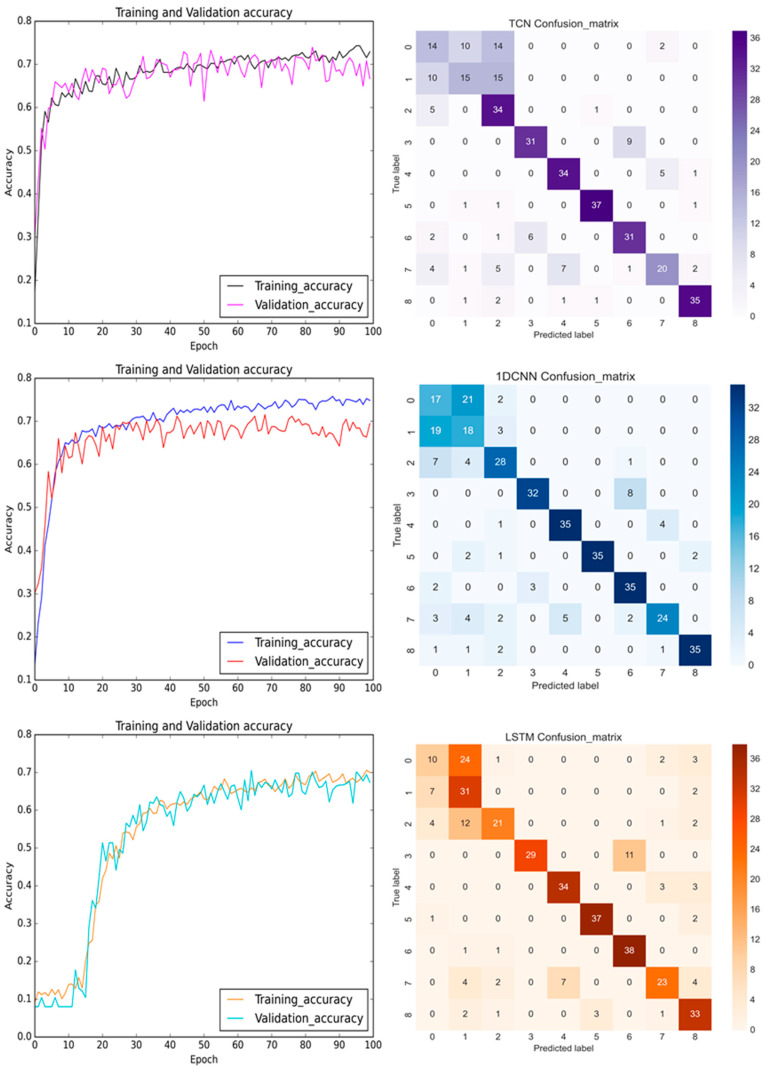
The training and validation accuracy with confusion matrix for TCN, 1D CNN, and LSTM (from **top** to **bottom**) before the milling process with the sensed signals in the *X*-axis.

Figure 17 shows the prediction results using the TCN, 1D CNN, and LSTM models for the pre-cutting of the sensed *X*-axis signals. A total of nine classes of healthy, damaged rotor, and damaged collet of DATH, with three different air pressures, 30 psi, 40 psi, and 50 psi are classified. As stated in Section 3.3.1, the top most and the second most affected signals were the sensed vibration signals in the *Z*-axis and the *Y*-axis, since the *Z*-axis (axial direction) was in the direction of the gravitational force on the DATH and the payload and a four-bar linkage planar motion with an elastic spring was in the Y-direction (the same as in the feed-drive direction). Therefore, the total features that were selected from the top five features of each class (0, 1, …, 8) were counted as 25 and 33 for the pre-cutting and after-cutting groups. The diversity of the features does not assist the training accuracy for the TCN, 1D CNN, or the LSTM prediction models. About a 73–74% training accuracy was obtained by the TCN and 1D CNN. Less than 70% prediction accuracy was displayed by the LSTM. The characteristics of the LSTM’s learning with time history showed a slow learning curve in the initial 10 epochs. In Figure 17, the lower prediction accuracies achieved by TCN, 1D CNN, and the LSTM in the confusion matrices were all in the normal 30 psi pressurized air. This may be caused by the lower excitation energy at 30 psi, and then, the feature extraction from free-running vibrational signals was not noticeable.

In Figure 17, the trends in the training and the validation accuracy improve in later epochs, since the weights of the NN were updated based on the same learning rate and the Adam optimizer. The neural networks of the TCN, 1D CNN, and the LSTM report all the bias and the weighting factors for each individual epoch. There is no guarantee of a monotonic increasing accuracy curve during the training process. Therefore, some oscillation in the epoch vs. accuracy graphs should be displayed heuristically. Nevertheless, the updated weights of the NN are used in the next epoch. This implies that the selection of the hyperparameters did a good job in improving the learning for the next epoch.

**Figure 18:**
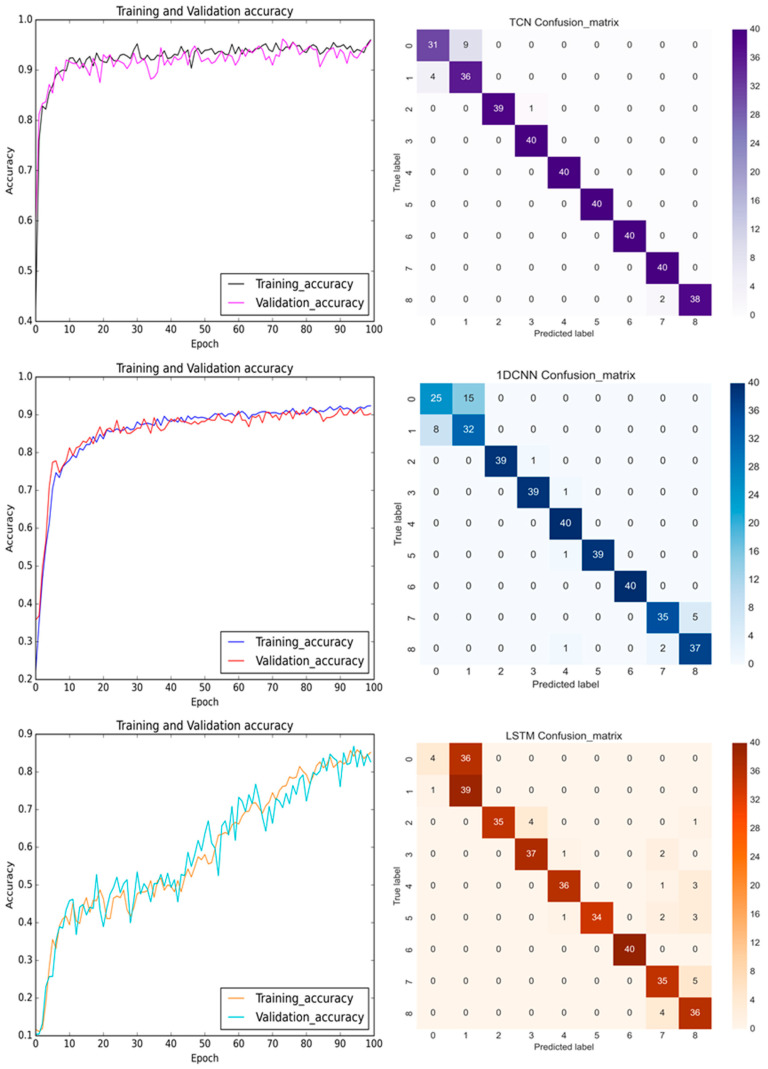
The training and validation accuracy with confusion matrix for TCN, 1D CNN, and LSTM (from **top** to **bottom**) before the milling process with the sensed signals in the *Y*-axis.

In the training process, Figure 18 shows the prediction results and confusion matrices for the TCN, 1D CNN, and LSTM models for the pre-cutting sensed *Y*-axis signals. The predictions of the TCN improved as the epoch time increased. The TCN converged quickly during the initial learning epochs. Since there were no pooling layers and the dilated causal convolution layers skipped the filter kernel over some neurons, the prediction accuracy oscillated through the epochs in Figure 18. The prediction accuracy of the TCN outperformed the accuracy of the 1D CNN and LSTM. This may be credited to the acquired vibrational signals being aligned with the feed-drive direction and the fixture being designed via a four-bar linkage planar motion with an elastic spring in the Y-direction. In Figure 18 (top), the confusion matrix of the TCN shows a convincingly better prediction accuracy.

**Figure 19:**
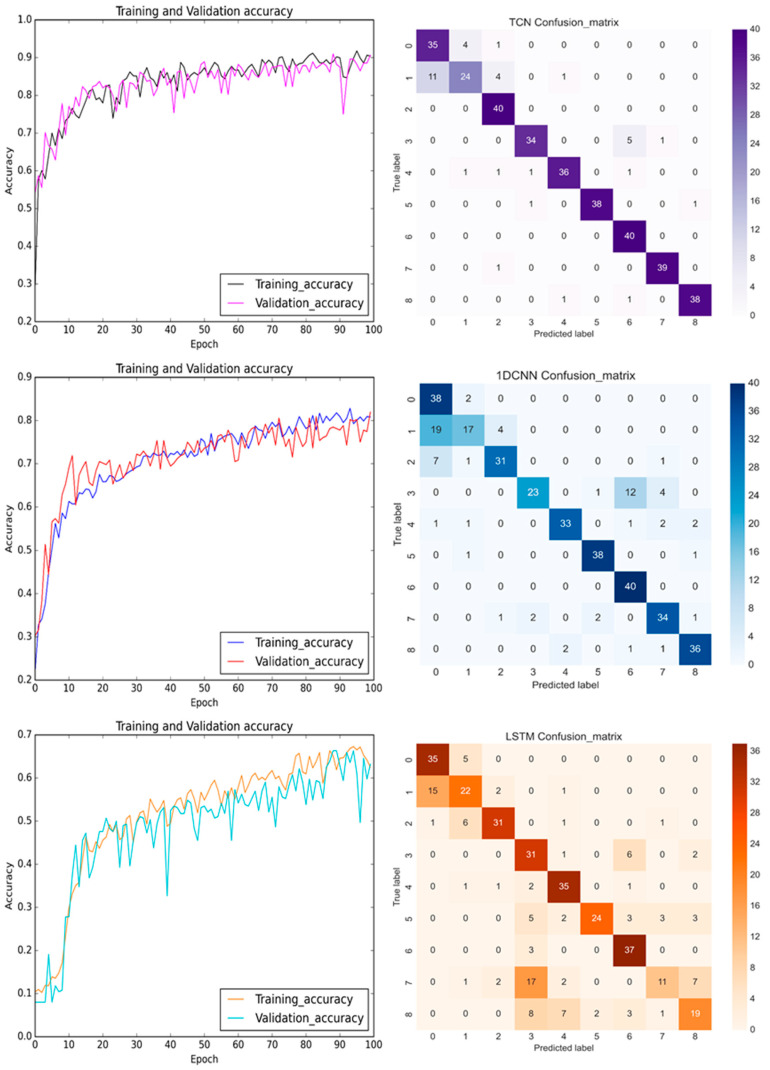
The training and validation accuracy with confusion matrix for TCN, 1D CNN, and LSTM (from **top** to **bottom**) before the milling process with the sensed signals in the *Z*-axis.

**Figure 20:**
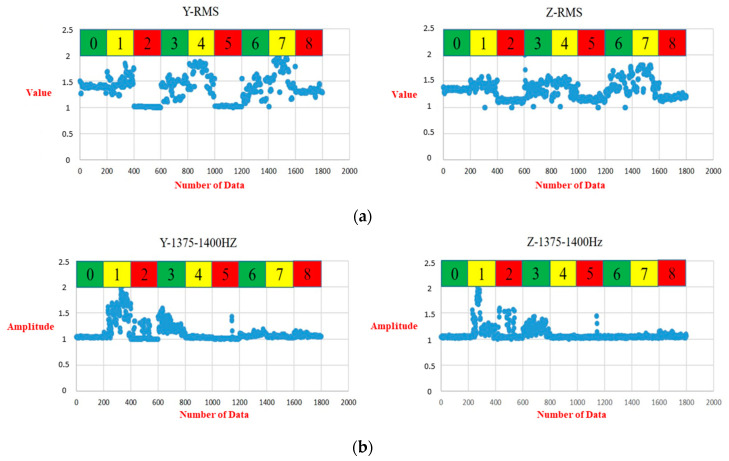
(**a**) Distribution of RMS (selected top feature in Table 4) for class 0 to 9 with sensed signals in the *Y*-axis (**left**) and *Z*-axis (**right**). (**b**) Distribution of the standard deviation (selected third-highest feature in Table 4) for class 0 to 9 with sensed signals in the *Y*-axis (**left**) and *Z*-axis (**right**).

In the training process, Figure 19 displays the prediction results and confusion matrices for the pre-cutting sensed *Z*-axis signals. Although the depicted plots of the prediction accuracy of the TCN in the *Z*-axis displayed more oscillation than those in the *Y*-axis, the overall model accuracy was still better than for the 1D CNN and the LSTM. Figure 20 details the distribution values of the RMS and standard deviation (STD) based on the top and third-top selected features in Table 4. The green, yellow, and red colors stand for the normal, damaged collet, and damaged bearing DATHs, respectively. The patterns of the RMS and frequency, ranging from 1375 Hz to 1400 Hz for the *Y*-axis and *Z*-axis are fairly alike. In conclusion, the featured information was used to establish a diagnostic health classification for the *Y*-axis and *Z*-axis. Table 6 shows the training and test accuracies of the TCN, 1D CNN, and LSTM with the selected input features. The developed TCN achieves the best prediction accuracy when the sensed vibrational signals were acquired from the *Y*-axis or the Z axis. Either the pre-cutting or the after-cutting signals can be used for diagnosing the health status of the DATH for dentists. The failure classification prediction model can accurately predict the health and failure mode of the dental handpiece before the use of the DATH when an embedded sensor or off-line sensing apparatus is available.

## 4. Discussion

### 4.1. The Advantages of TCN

As mentioned before, 1D CNNs have been used for fault identification and classification in many studies. The LSTM has demonstrated its capability to transfer memory and learn from time-series data. Since the heathy status of the DATH is a time-dependent case, the LSTM was used and compared with the TCN. The TCN, with the advantages for sequence modeling including parallelism, a flexible receptive field size, stable gradients, a low memory requirement for training, and variable-length inputs, was implemented successfully. The experimental results showed the TCN outperformed the 1D CNN and LSTM models in terms of prediction accuracy, especially in differentiating between healthy and damaged components of the DATH. Especially, based on the 40 psi and 50 psi tests, the TCN achieves the best prediction rate and outperforms the 1D CNN and LSTM.

### 4.2. The Limitations of the Proposed Method

In this study, feature engineering was adopted first. Selecting features improves the 1D CNN, LSTM, and TCN models’ predictions. In addition, in this research the number of neurons in the hidden layer, the number of epochs, and batch size were kept the same for each model. All the experimental results were based on the same learning processes till 100 epochs. The 1D CNN, LSTM, and TCN performed their independent neural work filter parameters or the said receptive field size parameters when the Adam optimizer were used and the learning rate was fixed as 0.001. As for the applications of neural networks, using feature engineering is not essential. All the raw data can be fed into the deep neural network no matter whether data cleaning was performed or not. However, the use of feature engineering proved to be a systematic way for the diagnostic processes in this study, since the experimental results showed notable advantages and improvements were observed with the TCN model in this study compared to the 1D CNN and LSTM approaches.

There is some potential for feature extraction using the continuous wavelet transform (CWT) [23] or the Hilbert–Huang transform (HHT) [24] for preparing training and labeled data for the deep learning (DL) models. The use of CWT and HHT with a CNN can be used for image decomposition by the characteristics of time and frequency spectra for feature classification. In future studies, the CNN could use CWT or HHT for image extraction.

## 5. Conclusions

The study’s conclusions are as follows:

1. This study employed a triaxial accelerometer to gauge the vibration signals of dental handpieces operating under unrestricted conditions. The collected data underwent feature engineering and selected features were the candidates for the input of the prediction model for diagnostic health classification.
2. The established temporal convolution network prediction model with the functions of causality in time sequence, transmitting memory, learning, storing, and fast convergence for monitoring the health and diagnosing the rotor and collet failure of DATHs, outperformed 1D CNN and LSTM models.
3. The experimental results revealed that use of the TCN framework generally provided better results than the use of a 1D CNN, which did not have a memory framework and had the drawback of the gradient vanishing problem. In addition, the TCN framework outperformed the LSTM model, which needed a long history to provide sufficient diagnostic ability. Still, high prediction accuracies were achieved by the TCN both in the direction of feed-drive milling and in the gravity of the handpiece through vibration signals.
4. The developed TCN model could accurately predict the health and failure mode of the dental handpiece before the use of the DATH when the embedded sensor or off-line sensing apparatus was available.
5. The experimental results show that pressurized air at 40 psi or more and a single accelerometer sensing in the direction of the *Y*-axis or *Z*-axis should be used.
6. There is potential for real-world applications and benefits of using the proposed predictive models for dental equipment maintenance and performance monitoring. If the vibrational MEMS sensor can be miniaturized and equipped with wireless fidelity or Bluetooth transmission technology, prognostic health management of the DATH in the future would be feasible.

## Figures and Tables

**Table 1:** Six time-domain features.

Feature	$\mathrm{Formula}\;(x_{i}\;as\;the\;Acquired\;Vibration\;Signal)$
RMS	$RMS=\sqrt{\frac{1}{N}\sum_{i=1}^{N}x_{i}^{2}}$
Variance	$VAR=\sigma^{2}=\frac{\sum_{i=1}^{N}{\left(x_{i}-u\right)}^{2}}{N}$
Standard Deviation	$\sigma =\sqrt{VAR}$
Skewness	$Skew=E[{\left(\frac{x-u}{\sigma }\right)}^{3}]$
Kurtosis	$Kurt=E[{\left(\frac{X-u}{\sigma }\right)}^{4}]$
Crest Factor	$CF=\frac{max\{x_{i},x_{i+1},\ldots ,\;x_{N}\}}{RMS}$

**Table 2:** The eight features calculated from the frequency-domain amplitudes for each section with a division every 25 Hz.

	PSD-Amplitude	PSD-Shape
Mean	$u_{amp}=\frac{1}{n}\sum_{i=1}^{n}C(i)$	$u_{shape}=\frac{1}{s}\sum_{i=1}^{n}iC(i)$
Standard Deviation	$\sigma_{amp}=\sqrt{\frac{1}{n}\sum_{i=1}^{n}{\left[C(i)-u_{amp}\right]}^{2}}$	$\sigma_{shape}=\sqrt{\frac{1}{s}\sum_{i=1}^{n}{\left[i-u_{shape}\right]}^{2}C(i)}$
Skewness	${sk}_{amp}=\frac{1}{n}\sum_{i=1}^{n}{\left[\frac{C(i)-u_{amp}}{\sigma_{amp}}\right]}^{3}$	${sk}_{shape}=\frac{1}{s}\sum_{i=1}^{n}{\left[\frac{i-u_{shape}}{\sigma_{shape}}\right]}^{3}C(i)$
Kurtosis	${ku}_{amp}=\frac{1}{n}\sum_{i=1}^{n}{\left[\frac{C(i)-u_{amp}}{\sigma_{amp}}\right]}^{4}-3$	${ku}_{shape}=\frac{1}{s}\sum_{i=1}^{n}{\left[\frac{i-u_{shape}}{\sigma_{shape}}\right]}^{4}C(i)-3$

*i*: index for the *i*th sector; C(i): the frequency amplitude; $s=\sum_{i=1}^{n}C\left(i\right)$: sum of the amplitudes in the *i*th sector, *n* = 25.

**Table 3:** Parameters of TCN, 1D CNN, and LSTM.

Parameter	TCN	1D CNN	LSTM
Residual Block	1	None	None
Dilation Rate	1, 2, 4	None	None
Neuron	50	50	50
Kernel_Size	2	2	None
Epoch	100	100	100
Batch_Size	32	32	32

**Table 4:** Number of the selected top 5 features for the *X*-axis, *Y*-axis, and *Z*-axis free-running signals based on comparing normal, damaged collet, damaged bearing, and normal DATHs at 30 psi, 40 psi, and 50 psi.

	N30 D30	D30 B30	N30 B30	N40 D40	D40 B40	N40 B40	N50 D50	D50 B50	N50 B50
*X*-axis	7	4	4 7	156	4	3	4	4	118
	143	49	76 83	157	84	55	164	118	120
	144	145	142 143	158	158	156	165	164 165	163
	145	146	144	159	159	157	166	166	164
	146	269	161	269	269	158	269	269	269
*Y*-axis	1	1	1	1	1	1	1	1	1
	3	2	2	3	2	2	3	3	2
	5	3	3	5	3	3	5	5	3
	59 62	5	5	64	5	5	66	70	5
	264	264	264	264	264	264	264	264	264
*Z*-axis	1	1	1	1	1	1	1	1	1
	3	2	2	3	2	2	3	2	2
	5	3	3	5	3	3	5	3	3
	62	5	5	67	5	5	70	5	5
	264	264	264	264	264	264	264	264	264

**Table 5:** Dataset for training, validation, and testing for classification of 9 classes.

Rotor Status_Air Pressure	Class	Number of Data Cells	Number of Training Data	Number of Validation Data	Number of Test Data
Normal_30Psi	0	200	128	32	40
Damaged Collet_30Psi	1	200	128	32	40
Damaged bearing_30Psi	2	200	128	32	40
Normal_40Psi	3	200	128	32	40
Damaged Collet_40Psi	4	200	128	32	40
Damaged bearing_40Psi	5	200	128	32	40
Normal_50Psi	6	200	128	32	40
Damaged Collet_50Psi	7	200	128	32	40
Damaged bearing_50Psi	8	200	128	32	40

**Table 6:** Training and test accuracy by TCN, 1D CNN, and LSTM with associated input features.

	TCN	1D CNN	LSTM	TCN	1D CNN	LSTM	Number of Input Features
	Training Accuracy			Test Accuracy			
X (pre-cutting)	74.51%	73.47%	68.97%	70.78%	70.44%	69.00%	25
Y (pre-cutting)	95.99%	92.03%	86.29%	94.83%	91.50%	86.11%	10
Z (pre-cutting)	88.88%	81.72%	68.08%	87.94%	79.28%	68.28%	8
X (after cutting)	71.85%	80.09%	64.81%	67.00%	77.61%	65.94%	33
Y (after cutting)	90.01%	84.10%	78.15%	89.00%	85.00%	78.61%	12
Z (after cutting)	90.82%	84.68%	76.99%	85.28%	82.56%	74.89%	9

## Data Availability

The vibrational raw data are available.
